# Editorial: Prediction of protein-protein interactions (PPIs): the next frontier

**DOI:** 10.3389/fmolb.2024.1479705

**Published:** 2024-08-26

**Authors:** Amar Singh, Binh P. Nguyen, Ho Leung Ng

**Affiliations:** ^1^ Computational Biology Program, The University of Kansas, Lawrence, KS, United States; ^2^ School of Mathematics and Statistics, Victoria University of Wellington, Wellington, New Zealand; ^3^ Department of Biochemistry and Molecular Biophysics, Kansas State University, Manhattan, KS, United States

**Keywords:** protein-protein interaction (PPI), protein-protein docking, homolog modeling, drug design, structural biology, molecular recognition

Protein-protein interactions (PPIs) are essential for biological processes, and their study is pivotal for elucidating cellular mechanisms and identifying potential drug targets. In recent years, the field of structural biology has seen remarkable advancements, particularly in modeling proteins’ tertiary and quaternary structures. The introduction of Artificial Intelligence (AI) and Deep Learning (DL) methods has shown a significant increase in accuracy for structure prediction. The recently released software AlphaFold3 (Abramson et al., 2024) and RoseTTAFold (Krishna et al., 2024) significantly expand these capabilities to a broader range of biomolecular interactions, setting a new standard for the prediction of PPIs. This Research Topic aimed to highlight promising advanced computational and experimental approaches for the prediction and characterization of PPIs. It includes original research contributions that delve into specific aspects of protein interactions and protein-protein recognition.

The first research article published in this Research Topic, authored by Shin et al., introduces PPI-Surfer, a novel computational method for comparing and quantifying similarities between PPI interfaces. The method captures both the 3D shape and physicochemical properties of protein surfaces using overlapping surface patches described by three-dimensional Zernike descriptors (3DZDs), allowing for fast comparison of molecular surfaces. Unlike alignment-based methods, PPI-Surfer is alignment-free and can identify local surface similarities in PPIs by exploring combinations of similar surface patches. To comprehend a variety of protein-protein interactions in a proteome, PPI-Surfer can be a valuable tool for discovering similarities, classifying PPI sites, and assisting in protein-based PPI drug discovery.

The Original Research article by Miller et al. investigated antibody-antigen complexes and identified several features that are crucial for distinguishing strong and weak antibody-antigen binding affinity. The developed machine learning models demonstrated good predictive performance by ranking the relative importance of different features, which include energetic, statistical, network-based, and machine-learned features, in determining binding affinity. The insights gained from this research could potentially expedite progress in understanding the structural features of antibodies, classifying antibody affinity, and designing and optimizing antibodies.

The following two research articles focus on interactions between specific domains of different proteins. The experimental study conducted by Genera et al. provides insight into the interactions and structural determinants of Protein Tyrosine Phosphatase Non-Receptor Type 3 (PTPN3). The focus of the research is on the PDZ domain of PTPN3, which is involved in PPIs, as well as its interactions with both viral and cellular partners. The *in silico* investigations by Shah et al. utilized epitope mapping techniques to identify specific regions on the SARS-CoV-2 Omicron subvariants that are targeted by neutralizing antibodies (nAbs). This study provides valuable insight into how these subvariants interact with antibodies, which is vital for improving vaccine and therapeutic strategies.

The Original Research article by Olatona et al. identifies proteins that interact with the cytoskeleton in basal airway epithelial cells *in vitro*. Advanced proteomics techniques, including mass spectrometry, and bioinformatic tools were employed to analyze the proteomics data, yielding a database of candidates that may link the structural elements.

In conclusion, the articles presented here highlight diverse computational and experimental approaches that, when combined, have the potential to significantly advance our understanding of specific protein interactions and proteomics as a whole.
